# Exploring tools used in the assessment of Trauma Team Leadership using video review: a systematic review

**DOI:** 10.1186/s13049-026-01643-z

**Published:** 2026-06-30

**Authors:** Cieran McKiernan, Prashant Kumar, Paul Bowie, David J. Lowe

**Affiliations:** 1 College of Medical, Veterinary and Life Sciences, University of Glasgow, Glasgow, UK; 2 Queen Elizabeth University Hospital, NHS Greater Glasgow and Clyde, Glasgow, UK; 3 NHS Education for Scotland, Glasgow, UK; 4 HealthTech Innovation and Translation Lab, University of Glasgow, Glasgow, UK

**Keywords:** Trauma team leadership, Video review, Non-technical skills

## Abstract

**Background:**

Leadership is a key non‑technical determinant of trauma team performance and is associated with adherence to protocols and patient outcomes. Retrospective video review is increasingly used to evaluate trauma resuscitations; However, the structured observational tools used to evaluate trauma team leadership during video review are heterogeneous and inconsistently reported. This systematic review identifies and characterises structured observational tools used during video review to assess trauma team leadership behaviours.

**Methods:**

This systematic review was conducted in accordance with PRISMA 2020 guidelines and prospectively registered on PROSPERO (CRD42025636807). MEDLINE, Embase and CINAHL were searched without date restrictions. Studies were eligible if they used retrospective video review of real or simulated trauma resuscitations and applied a structured observational tool to assess leadership or leadership‑related non‑technical skills. Two reviewers independently screened studies extracted data and performed methodological appraisal using the Mixed Methods Appraisal Tool (MMAT). Due to substantial heterogeneity, results were synthesised narratively.

**Results:**

Twenty studies published between 1988 and 2024 were included. Most studies were conducted in the United States (*n* = 15), with additional studies from Canada (*n* = 3) and the Netherlands (*n* = 2). Nineteen distinct structured assessment tools were identified. The Non‑Technical Skills Scale for Trauma (T‑NOTECHS) was the most frequently used instrument (*n* = 7), followed by the Mayo High Performance Teamwork Scale and the EM Resuscitation Team Leadership Measure. Considerable variation existed in leadership domains assessed, scale structure, rater background and rater training. Communication, decision‑making, task management and situational awareness were the most commonly evaluated leadership attributes. Inter‑rater reliability was inconsistently reported and ranged from poor to excellent, with higher agreement generally observed in simulation‑based assessments.

**Conclusions:**

Multiple tools have been used to assess trauma team leadership using video review, with marked heterogeneity in design, rater approach, and reliability reporting. While T-NOTECHS is the most commonly applied instrument, variation in constructs and scoring limits comparability across studies and trauma centres and complicates evaluation of leadership-focused education and quality-improvement initiatives. Inconsistent reliability evidence also constrains outcome-linked research. Greater consistency in leadership assessment tools, rater training, and psychometric reporting is therefore required to support benchmarking, robust evaluation of improvement programmes, and research linking leadership behaviours to clinical and system outcomes.

**Supplementary Information:**

The online version contains supplementary material available at https://doi.org/10.1186/s13049-026-01643-z.

## Introduction

Major trauma is defined as “an injury or combination of injuries that are life-threatening and could be life-changing due to long-term disability.” [1] Major trauma remains the leading cause of death and disability in people under 45 worldwide and is responsible for nearly six million deaths annually [2], accounting for approximately 10% of all global mortality [3]. The European Union accounts for 140,000 of these fatalities [4]. Survivors often experience significant physical, psychological, and social sequelae [5]. Effective trauma management is therefore critical, as early recognition of life-threatening injuries, rapid decision-making, and coordinated multidisciplinary care are key determinants of both survival and long-term functional outcomes.The heterogeneous nature of trauma with variation in causes, severity, and clinical presentation pose substantial challenges to prognostication and clinical management [6].

Trauma teams are utilised across a wide range of health systems and hospital settings and involve designated team leaders to coordinate multidisciplinary care. Internationally, many trauma systems have undergone regionalisation, with a substantial proportion of the published literature originating from Major Trauma Centres (MTCs). These centres are typically staffed by consultant‑led multidisciplinary teams with higher numbers of major trauma presentations and have been associated with improved patient outcomes, including reductions in mortality and improved functional outcomes, compared with non‑specialised hospitals [7]. Within these and other trauma care settings, trauma teams comprise clinicians from multiple specialties, including emergency medicine, surgery, anaesthesia, and intensive care, who must work collaboratively under dynamic and time‑critical conditions. While MTCs represent the predominant study setting within the existing evidence base, the leadership constructs examined are relevant across all trauma teams. These “flash teams” are rapidly assembled in response to acute trauma presentations, often with minimal preparation time and dynamic, non-standardised team configurations. Multiple patients may also arrive simultaneously or in rapid succession, placing additional demands on both the system and the clinical team. In response to these operational challenges, healthcare has adopted principles from aviation-derived Crew Resource Management (CRM), which emphasise non-technical skills such as communication, stress management, decision-making, fatigue management, leadership, situational awareness and teamwork to optimise patient care [8]. The importance of these skills has been demonstrated in many studies involving trauma resuscitations [9, 10], and has led to the widespread adoption of these principles with educational trauma courses, such as Advanced Trauma Life Support (ATLS) and Pre-Hospital Trauma Life Support (PHTLS), worldwide.

Several leadership styles are described in resuscitation teams, from ‘Lighthouse Leadership’ [11] which emphasises guiding and directing resuscitation teams from a distance, establishing structure, and maintaining control through ‘Adaptive’ [12] or ‘Contingent’ [13] leadership”, which involves dynamically adjusting leadership functions or roles, to ‘Democratic leadership [14] which emphasizes collaboration and inclusivity, encouraging team members to contribute their opinions and participate in decision-making processes. Regardless of the method, the trauma team leader (TTL) plays a pivotal role in directing the resuscitation, maintaining situational awareness, and ensuring efficient task allocation [15, 16]. During High Acuity, Low Occurrence (HALO) trauma resuscitations, the skills required for this role are of paramount importance. Communication has been consistently reported as a critical leadership skill in numerous medical action teams and was identified as the most significant attribute defining excellence in a trauma team [17]. The TTL is the driving force for the NTS application within the team.

Retrospective video review has emerged as a valuable tool for analysing trauma resuscitations. It has long been used for simulation debriefing, providing re-playable data for post-event evaluation, reducing recall bias, and enhancing team learning that may not be apparent through memory based review alone [18, 19]. However, despite the potential, tools used to assess leadership through this type of video review vary widely in their conceptual foundations, domains of assessment, measurement approaches and reporting of validity or reliability. This methodological variability limits comparability across studies and inhibits consistent application within trauma education and quality improvement. To address this gap this systematic review aims to identify and describe observational assessment tools applied during video review to evaluate trauma team leadership, mapping tool characteristics, leadership constructs assessed, application contexts, and reliability reporting, with the intention of informing future use in education, simulation and trauma quality improvement systems. The research question is, ‘*What structured observational tools are used during retrospective video review to assess trauma team leadership?*

## Methods

### Study design

This systematic review was conducted and reported in line with PRISMA 2020 guidance [20] and was prospectively registered on PROSPERO (CRD42025636807).The research question was structured using the PICOS framework to guide eligibility criteria, searching, and data extraction [21] (Table 1).

**Table 1:** PICOS framework used to construct research question

Element	Description
Population	Trauma teams or resuscitation teams in emergency or simulation settings
Intervention / Exposure	Use of *video review* (real or simulated)
Outcome	Assessment of *team leadership* or leadership-related *non-technical skills* (NTS)
Study Design	Observational studies, simulation studies, RCTs, educational interventions both qualitative and quantitative

### Search strategy

Finding the relevant literature involved using a variety of vocabulary and terms to cover the range of conceptual descriptors, such as assess* and evaluate* and methods of this review including tools* and taxonomy*. Syntaxes, parentheses and Boolean operators were used. Full search terms can be seen in detail in Appendix 1. Using these search criteria, the author conducted a review of the MEDLINE, Embase and CINAHL databases. Reference lists and grey literature were also screened. No limitation in publication date was used within the search criteria. By not limiting the publication date for the literature search we were endeavouring not to miss any study within such a specialised field with presumed limited amount of research.

### Eligibility criteria

Studies were eligible if they met all of the following criteria:

- Used retrospective video review of real or simulated trauma resuscitation events;
- Applied a structured observational assessment tool (Likert-type and/or behaviourally anchored) to evaluate leadership or leadership-related non-technical skills (NTS).
- Were peer-reviewed and published in English.

We excluded qualitative discourse/content analyses without a rating tool, technical-only checklists (e.g., primary survey completion forms without behavioural leadership assessment), editorials/letters/opinion pieces/conference abstracts, and non-trauma contexts.

### Study selection and data extraction

All duplicate records were removed and screened in two stages (title/abstract, then full text). Titles and abstracts were screened for inclusion if they contained the words, trauma, video, lead(er) or any of the chosen synonyms for assessment. The identified papers were transferred into *Covidence (Veritas Health Innovation Ltd.*,* Victoria*,* Australia)* [22]. 

**Fig. 1:**
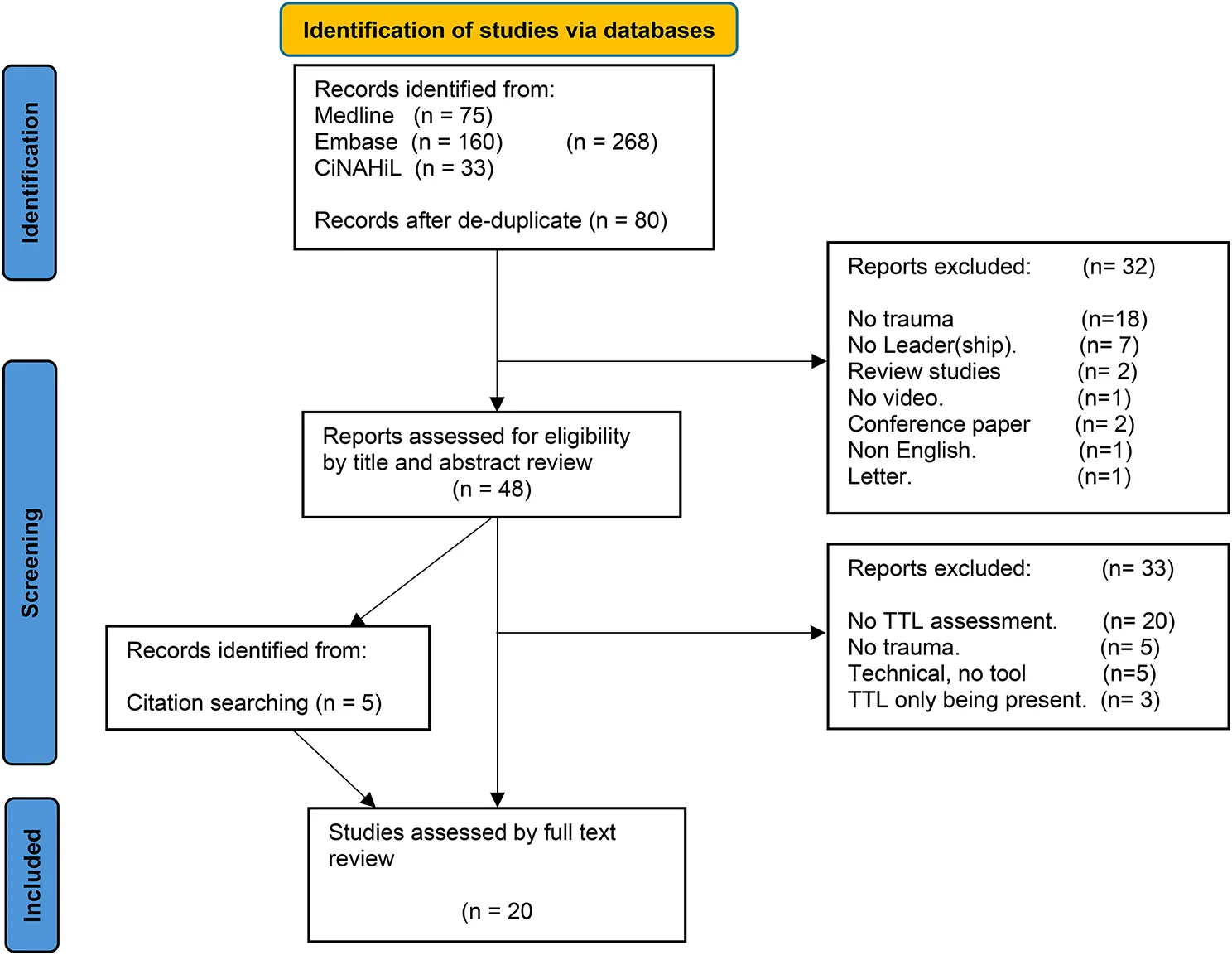
Flow chart of process of selection of included studies

Data extracted included study characteristics (year, country, setting—simulation/live, adult/paediatric), population and team configuration, assessment tool (name, domains, scale, any modification), leadership constructs, rater number/background/training, inter-rater reliability statistics (e.g., κ, ICC, Gwet’s AC2, PABAK), and key results related to leadership-related NTS. All screening and full-text data extraction was carried out by two independent reviewers, the lead author CMcK and PK. Any disagreements between the reviewers at each stage of the selection process were resolved through discussion with an additional independent third reviewer DJL The search results and the study inclusion process are reported in a Preferred Reporting Items from The PRISMA 2020 statement: an updated guideline for reporting systematic reviews flow diagram (Fig. 1).

### Data evaluation

Methodological appraisal was performed using the Mixed Methods Appraisal Tool (MMAT). This tool appraises quality within a range of study types which previously required different criteria to demonstrated quality. including direct observational and experimental studies that can be either quantitative, qualitative or mixed methods in their methodology [23]. The tool uses five different criteria across each design type which allows for comparison of criteria met by different study types. If one of the quality criteria are met, then the designers of the tool suggest a quality score of 20% increasing proportionally up to five criteria being met to give a score of 100%. Study heterogeneity prevented any form of meta-analyses being undertaken so findings were synthesised narratively.

**Table 2:** Key Characteristics of studies assessing trauma team leadership using structured observational tools

		First Author Year Country	Type of study	Sample Size Team Configuration	Tool Name Scoring Range	No of Raters	Experience of Raters	Reliability of rating IRR
Study Setting	Simulation (all adult)	Briggs [24] 2015 USA	Retrospective cohort study	20 teams case 1 15 teams case 2 Not defined in number	Non-Technical Skills for Surgeons (NOTSS Likert 1–4 For leader Non-Technical Skills Scale for Trauma (T-NOTECHS) Likert 1–5 For team	2 2	One attending physician. One postgraduate year 3 (PGY3) resident One attending physician. One postgraduate year 3 (PGY3) resident	NOTSS 0.83 NTSs performance by TTL in all categories decreased during scenario ( all *p* < 0.05). T-NOTECHS 0.4 (0.49–0.69 each category)
		Crozier [25] 2014 Canada	Prospective observational study Traditional checklist (ATLS) assessed using retrospective video	4 teams case 1 4 teams case 1 3 members 12 participants 4 Teams, one each student, junior, senior, attending	(Team) Situation Awareness Global Assessment Technique ((T)SAGAT) Total score: 100 points 50 competencies in 7 categories Likert 0–2	2	Not defined	Pearson correlation coefficient of *r* = 0.937. Compared checklist with tool not different raters
		Fernandez [26] 2019 USA	Single-blinded randomized controlled trial	60 participants (30 in intervention group and 30 in control group) Not defined in number	Team Leadership Behavioural Measure 5 Domains Different score range in each Maximum score 38 points	Not described	Trained but otherwise not defined	Ranged from 0.9 to 1.0 for individual items in the leadership measure,
		Hamilton [27] 2012 USA	Prospective intervention study	11 PGY-2 general surgery and GS and plastics combined 4 member team, leader, primary survey, procedure, airway 2 sessions with 4 scenarios each	Mayo High Performance Teamwork Scale (Modified for Trauma)	28	15 Clinicians Attending surgeons PGY4/5 surgery Experienced trauma Nurses 13 Non-clinicians Undergraduate business students	No IRR for leadership Overall IRR for team evaluation, given as an intraclass correlation coefficient (ICC) of **0.926.**
		Leenstra [28] 2021 Netherlands	Development and evaluation of the taxonom. Two-phase iterative testing Phase 1:video-based testing Phase 2: live testing during ATLS refresher courses.	32 participants across both phases.	TTLS-SHORT. Taxonomy of Trauma Leadership Skills–Shortened for Observation and Reflection in Training 3-point Likert scale	31 Phase1: 10 raters Phase 2: 21 raters	Trauma care experts and instructors with experience in training NTS. Various professions and seniority Experience < 1 year to over 22 years. Full details given in paper	No statistical measure Mentions steps taken to ensure the reliability and validity of the tool and the raters’ evaluations
		Lorello [29] 2016 Canada	Prospective, single-blinded, randomized study.	78 PGY1-5 2 member team 19 teams in the control group 20 teams in the mental practice (MP) group	Mayo High Performance Teamwork Scale (MHPTS) 3-point Likert scale	2	Expertise in teamwork and trauma.	IRR given as an intraclass correlation coefficient (ICC) of **0.6.**
		Ortiz Figueroa [30] 2016 USA	Prospective feasibility study	15 interns various specialties: Surgery (4) Orthopaedics (7) Urology (2) EM (2)	T-NOTECHs Likert 1–5	3	Trained ATLS instructors	No statistical measure
		Rosser [31] 2023, USA	Quantitative experimental	150 5 member team 30 teams	T-NOTECHs Likert 1–5	3	Faculty members Trauma surgery, EM, nursing	≥ 0.8. between two human raters and the corresponding automated coding algorithms
	Real	Bhangu [32] 2022 Canada	Quality improvement study following SQUIRE 2.0 guidelines	55 trauma activations 9 member team Adult	T-NOTECHs Likert 1–5	2	A medical student and emergency department nurse. IRR 0.52	Measured using the intraclass correlation coefficient (ICC), was 0.52 for the
		Dumas [33] 2022, USA	Descriptive study on Trauma Video Review (TVR) programs	Not specified Team size not defined Adult	T-NOTECHs Likert 1–5	2	Attending trauma surgeon Resident	Not mentioned
		El-Shafy [34] 2018 USA	Prospective observational study	89 trauma 7 team members Paediatrics	Team STEPPS Likert 1–5	2	Not specified	Not mentioned
		Hartwell [35] 2024 USA	Prospective cohort study	146 videos reviewed involving 12 residents 10–14 team members Paediatrics	Novel team leadership assessment tool 5-point Likert scale,	3	A simulation specialist, a clinical staff paediatrician, a PGY-3 Paediatric resident	Gwet’s agreement coefficient (AC2) used to evaluate scores among the reviewers for each domain of the Team Leadership Assessment Tool. For individual domains 0.45 to 0.59. Total score having an 0.49. All reliability values were statistically significant (*p* < 0.0001).
		Henkel [36] 2016 USA	Mixed-methods study	30 traumas 12 PEM leads 11–12 team members Paediatrics	T-NOTECHs Likert 1–5	3	PEM physicians, Paediatric surgeons Non-physicians (nurses and advanced practice (APPs)).	Fleiss’ Kappa: summed 0.02 leadership strongest 0.25 ICC 0.27–0.55 PEM vs. Surgeon: ICC = 0.50 Surgeon vs. RN/APP: ICC = 0.27 PEM vs. RN/APP: ICC = 0.55 PEM vs. PEM self-review: ICC = 0.49
		Hoff [37] 1997 USA	Retrospective observational study	425 traumas 9 member team Adult	No tool name Used a performance improvement audit form both objective and subjective	3	Attending Traumatologist Trauma Fellow (PGY-6/PGY-7) Trauma Nurse	Not mentioned
		Hoyt [38] 1988 USA	Prospective observational study	180 traumas in study group, 60 in control Team size not specified Adult	No tool name A videotape audit form	2	Trauma attending physician Trauma nurse co-ordinator	Not mentioned
		Lubbert [39] 2008 Netherlands	Retrospective observational study	387 traumas 6 team members Adult	No tool name Standardised score sheet	1	Attending surgeon	Not measured
		Maiga et al [40] 2016 USA	Retrospective analysis of a prospective multicentre observational study	441 patient Team members Median 12 IQR 10–15 Adult	T-NOTECHs (modified) Likert 1–3	1–3	Not mentioned Each had 2 training sessions	0.71 for TVR of resuscitations
		Rosenman [41] 2019 USA	Pilot study	10 traumas No specific team size Adult	EM Resuscitation Team Leadership Measure Likert 1–5	4	Board-certified Emergency Medicine (EM) physicians:	Used ICC For all observations: 0.79 For simulation-based observations: 0.87 (medical cases) For actual patient care observations: 0.24 (trauma cases)
		Rosenman [42] 2021 USA	Secondary analysis of data from Rosenmann 2019	60 leaders 2 traumas each 120 recordings Adult	EM Resuscitation Team Leadership Measure Likert 1–5	4	Board-certified Emergency Medicine (EM) physicians:	The probability and bias-adjusted kappa (PABAK) was calculated to adjust for prevalence, as the measure targeted low base rate events. The mean PABAK was 0.97 across all items,
		Steinman [43] 2016 USA	Prospective observational study	244 traumas 103 post training 5 or 10 member team (modified or full) Adult	T-NOTECHs Likert 1–5	17	13 critical care trauma nurses (CRN) 4 research assistants (3 medical students and 1 physician).	ICC used to measure between CRN and research assistants 0.48

## Results

A total of 268 records were identified across databases: after removing duplicates,

80 remained. Following title and abstract screening, 48 full texts were assessed, of which 15 met eligibility criteria. An additional five studies were identified through citation searching, resulting in 20 included studies.

## Study characteristics

Included studies were published between 1988 and 2024. The key characteristics of the included studies are detailed in Table 2. 11 studies achieved 100% [26, 28, 29, 31–35, 38, 40, 43] in the quality review, 8 studies scored 80% [4, 25, 27, 35–37, 39, 40–42] with 1 study scoring only 40% [30]. 15 studies were conducted in the USA (75%), [24, 26, 27, 30, 31, 33–38, 40–43] three in Canada [25, 29, 31], and two in the Netherlands [28, 39]. The team leaders being assessed are from a range of different specialities in each study, and this varied by country. The USA based studies reported leaders as either surgeons or emergency physicians. In studies from the Netherlands, surgical residents were designated as leaders [28, 39]. The grade of the leader within the clinical hierarchy was variable throughout the studies. Eleven studies did not comment on the grade of the leaders. [24, 28, 30–35, 38, 39, 43] Fernandez [26] recruited 79 s- and third-year residents, Hamilton [27] assessed 11 post graduate year residents without mentioning which year, Hartwell [35] reviewed 12 fellows while Lorello [29] enrolled 78 postgraduate trainees from resident years 1 through 5. There was also no standardisation of trauma team size being led with only some simulation studies specifying number of team members ranging from 2 [26] to 14 [36].

### Study environment

Three studies were undertaken in a paediatric only trauma centre [34–36], the remainder were not specific with regards to the inclusion or number of paediatric patients included in their studies. Twelve of the included studies used guidelines and teaching from ATLS training courses either as a standard to measure performance against or as a marker of ability to be a trauma team leader [26–29, 31, 32, 35, 36, 38–42]. Eight of the studies were set in the simulation environment [24–31], with the others being set in real patient presentations to trauma centres. Rosenman et al. [41] used a mix of simulated (10) and real (20) cases. For this systematic review, the real cases were included as they were trauma-related however the simulated case involved resuscitating respiratory distress progressing to cardiac arrest so were excluded.

### Trauma severity

Seven studies reported injury severity using the Injury Severity Score (ISS), a widely used measure quantifying overall trauma severity on a scale from 1 to 75 [44]. Within this context, ISS provides an indicator of case complexity, with higher scores typically reflecting greater physiological compromise, more complex decision-making, and increased demands on team coordination. Consequently, ISS may act as a proxy for resuscitation complexity, enabling interpretation of leadership performance relative to the clinical challenges faced by the trauma team. As the studies describe importance of team leader in patient outcomes, associating these presentations with degree of injury severity could be an appropriate research aim. Eight of the studies describe ISS scoring of their patients [27, 29, 33, 37, 38, 40–42], but none use a consistent structure of measurement. Leenstra [28] used > 15 as the point of major trauma classification with 260 patients, out of 3459 being in this group. Fernandez [26] mentions ISS but gives no further expansion.

### Assessment tools

Across the 20 included studies, the most frequently used leadership-related NTS tool was T-NOTECHS (*n* = 7; 35%). Other instruments were described in two studies each (10%), EM Resuscitation Team Leadership Measure and Mayo High Performance Teamwork Scale (MHPTS). Single studies used NOTSS, TSAGAT, TTLS SHORT, TeamSTEPPS, the Team Leadership Behavioural Measure, and a novel paediatric leadership tool (each *n* = 1; 5%). Three studies used local audit or scoring systems (e.g., centre-specific video audit forms) to observe leadership with task performance (*n* = 3; 15%). Full counts are summarised in Table 3.

**Table 3:** Assessment tools

Assessment Tool	Number
Non-Technical Skills Scale for Trauma (T-NOTECHS)	5
Modified Non-Technical Skills Scale for Trauma (T-NOTECHS)	2
Mayo High Performance Teamwork Scale (MHPTS)	2
EM Resuscitation Team Leadership Measure	2
Team Situation Awareness Global Assessment Technique (TSAGAT)	1
Traditional Checklist	1
Team Leadership Behavioural Measure Domains	1
Non-Technical Skills for Surgeons (NOTSS)	1
TTLS-SHORT (Taxonomy of Trauma Leadership Skills–Shortened for Observation and Reflection in Training)	1
Team STEPPS	1
Novel team leadership assessment tool (part based on MHPTS)	1

### Leadership attributes assessed

Early studies assessing trauma team leadership used observational approaches. Hoyt (1988) [38] evaluated leadership performance through video review of trauma resuscitations, using the proportion of “wasted time” as an indicator of performance. In the intervention group exposed to video-based review, wasted time decreased from 37% to 15% among patients with matched Injury Severity Scores, compared with a smaller reduction in the control group. Subsequent studies applied structured and behaviourally anchored assessment frameworks. The Non-Technical Skills for Surgeons (NOTSS) system assessed leadership across four domains comprising 12 elements, each rated on a four-point Likert scale [25]. Rosenman et al. developed a team leadership assessment tool consisting of 15 items based on behaviourally anchored rating scales (BARS), organised into six leadership dimensions, each scored on a five-point Likert scale [42]. Hartwell et al. evaluated six leadership elements using a five-point Likert scale within an adapted team assessment tool [36]. The identification of over 100 distinct leadership attributes highlights the absence of a unified construct of trauma team leadership.The most frequently reported attributes are summarised in Table 3. Each attribute was counted once per assessment tool to avoid overrepresentation (Table 4).

**Table 4:** Most common leadership attributes assessed

Leadership Attribute	Frequency	Mentions
Communication	6	Communication, Explicit Communication, Communication Skills, Effective Communication
Decision-Making	5	Decision-Making
Task Management	4	Task Delegation and Role Adherence, Task Assignment, Task Management, Task Coordination
Situation Awareness	4	Situation Awareness
Team Collaboration	3	Promotes Team Collaboration, Team Direction, Team Involvement
Role Clarity	3	Role Delegation, Role Clarity, Adherence to Responsibilities

### Reliability reported across studies

Rater backgrounds varied across studies and included attending surgeons, emergency physicians, nurses/advanced practice providers, residents/fellows, and trained non-clinicians. Rater training ranged from brief calibration exercises to structured training programmes linked to specific tools. Inter-rater reliability (IRR) was reported using multiple statistical methods. Cohen’s Kappa (κ) was the most frequently used measure (*n* = 8), with reported values ranging from 0.4 to ≥ 0.8 [25, 34]. Intraclass Correlation Coefficients (ICC) were reported in two studies, with values of 0.27 and 0.48 [37, 43]. Gwet’s Agreement Coefficient (AC2) was used in one study, with domain-level agreement ranging from 0.45 to 0.59 and a total score of 0.49 demonstrating moderate agreement [36]. Fleiss’ Kappa was reported in one study, assessing three raters using T-NOTECHS with an overall value of 0.02 and a maximum of 0.25 within the leadership domain [37]. The probability and bias-adjusted Kappa (PABAK) was reported in one study, with a mean value of 0.97 across items, indicating almost perfect agreement with adjustment for bias and prevalence [42]. Reliability reporting was incomplete in a subset of studies (*n* = 7;35%), limiting cross tool comparability and meta-analysis.

## Discussion

This review represents the first systematic synthesis of studies assessing trauma team leadership-related non-technical skills (NTS) using retrospective video review. While broadly consistent with the scoping review by Bhangu [45], this analysis is focused, examining structured tools used specifically for leadership assessment within video review. The increase in included studies (12 vs. 20) reflects growing recognition of leadership as a critical non-technical determinant of trauma team performance. This review identifies (i) the range of tools used to assess leadership behaviours, (ii) the leadership attributes they evaluate, and (iii) the variability in inter-rater reliability (IRR) reporting across simulation and clinical settings.

Retrospective video review emerged as a feasible and increasingly utilised method for assessing trauma team leadership. Compared with real-time observation, it enables replay, structured scoring, and independent multi-rater assessment, supporting more consistent evaluation and feedback. Similar benefits have been demonstrated in other high-risk clinical settings, including obstetrics and gynaecology [46], suggesting broader applicability across acute care environments. T-NOTECHS was the most frequently used assessment tool. Its widespread use likely reflects its derivation from the NOTECHS framework and its ability to assess leadership within a broader non-technical skills construct, including communication, cooperation, situational awareness, and decision-making. Its relatively simple structure and ease of application make it suitable for high-acuity environments [47]. In contrast, more granular tools may offer detailed behavioural assessment but are less practical for routine clinical or quality improvement applications.

Communication was the most consistently assessed leadership attribute. This included clarity of communication [27, 31, 38, 43] and use of closed-loop communication [37], reflecting its central role in coordinating team activity and maintaining shared situational awareness. While individual leadership behaviours were frequently evaluated, few studies examined how these behaviours combine to form broader leadership styles. Previous work by Jacobsson suggests that trauma team leaders adapt communication strategies and repertoires to situational demands [48], highlighting an important gap in current measurement approaches.

Despite the availability of structured tools, substantial variability in inter-rater reliability was observed. Differences in rater background, training, and assessment context contributed to this variation. Reliability tended to be higher in simulation settings, where environmental complexity is reduced and behavioural performance is more stable, and when assessments were conducted by experienced raters, suggesting that both context and assessor expertise materially influence scoring consistency. In contrast, real-world trauma environments introduce competing stimuli, interruptions, and parallel task demands, which may reduce behavioural observability and attenuate agreement between raters. Variation in statistical measures of reliability, with differing thresholds used to define agreement between raters, limited comparability across studies and precluded meta-analysis. In addition, reporting of validity was limited, and few studies evaluated whether tools accurately capture leadership behaviours associated with improved team performance or patient outcomes.

Variation in scale design also contributed to heterogeneity. Likert scales ranged from three to five points, reflecting a balance between usability and sensitivity [49]. Although five-point scales are commonly used, variation in scoring systems limited comparability across studies and precluded meta-analysis.

Overall, there remains a lack of standardisation in the assessment of trauma team leadership. Future research should focus on validating leadership-specific assessment tools in real clinical environments, standardising rater training, and improving reporting of psychometric properties, particularly reliability and construct validity. Real-world evaluation is essential to determine whether observed leadership behaviours translate into measurable clinical and system outcomes. Studies are required to support development of standardised frameworks applicable across trauma education, and quality improvement programmes.

### Strengths and limitations

This systematic review has several strengths. To our knowledge, it is the first to synthesise tools specifically used for video-based assessment of trauma team leadership, rather than broader non-technical skills or teamwork frameworks. The review was conducted using robust methodology, including a multi-database search strategy, dual independent screening, structured data extraction, and formal quality appraisal using the Mixed Methods Appraisal Tool (MMAT) [23]. Prospective registration on PROSPERO further enhanced transparency and methodological rigour. In addition, the review identifies key assessment tools (e.g., T-NOTECHS, EM Resuscitation Team Leadership Measure, MHPTS, TeamSTEPPS) and summarises the primary leadership attributes assessed, supporting potential application in education, simulation, and trauma governance.

Several limitations should be considered. First, the review was restricted to English-language publications, introducing potential language and publication bias and the possibility that relevant tools were not identified. Second, although broad search terms were used, variation in terminology may have resulted in the omission of relevant studies. Third, substantial heterogeneity was observed across included studies in terms of design, clinical context, team configuration, leadership constructs, rater background, and assessment tools, which precluded meta-analysis and necessitated a narrative synthesis. Finally, reporting of psychometric properties was inconsistent, particularly with respect to inter-rater reliability and rater training, limiting comparison between tools and restricting conclusions regarding their relative validity and performance.

### Implications of heterogeneity

The heterogeneity identified in this review has important implications for trauma centres using video review for education or quality improvement. Differences in constructs measured (e.g. leadership versus broader teamwork), behavioural anchors, scoring granularity, and observation frameworks, mean that observed “performance” may be influenced as much by instrument choice as by true differences in team behaviour. This limits interpretability of local audit data and complicates selection of tools aligned with intended use, whether formative feedback, summative assessment, or system learning. Heterogeneity also increases implementation burden, as tools require different levels of rater training and calibration, reducing scalability of routine video-review programmes.

A further implication is reduced comparability between centres and across time. Without greater standardisation, it is challenging to benchmark leadership and subsequently team performance across trauma systems, or to aggregate findings across studies, because scores may not represent equivalent constructs. This also limits rigorous evaluation of performance-improvement initiatives (e.g., leadership training courses or structured video debriefing), as observed change may reflect differences in measurement rather than true improvement. Even within a single centre, switching between tools for different leaders can disrupt longitudinal measurement and dilute any signs of improvement.

Finally, inconsistent reporting of inter-rater reliability has direct implications for research linking leadership behaviours to process or patient outcomes. Measurement error arising from poor or unknown inter-rater reliability can attenuate true associations and lead to unstable or non-reproducible findings, particularly in large MTC centre studies where raters vary. Reliability is therefore a prerequisite for valid interpretation of differing leadership performances. While some tools may be suitable for formative educational use, stronger and more consistently reported psychometric evidence is required before leadership ratings can be used for benchmarking or outcome-linked research.

## Conclusion

Video review represents a potentially powerful mechanism for evaluating and improving trauma team leadership. This review demonstrates substantial heterogeneity in the observational tools used to assess leadership during trauma video review, including variation in constructs assessed, scoring approaches, rater background and training, and reliability reporting. This matters because such variability limits meaningful comparison across centres and studies, complicates evaluation of leadership-focused education and quality-improvement initiatives, and weakens outcome-linked research when measurement reliability is unclear or inadequate. Moving toward greater consistency—through clearer construct definitions, agreed core domains, standardised scoring anchors, and routine reporting of reliability with transparent rater training—would support benchmarking across trauma systems, more defensible evaluation of training and debriefing interventions, and stronger multicentre studies examining links between leadership behaviours, team performance, and patient outcomes.

## Supplementary Information

Below is the link to the electronic supplementary material.

Supplementary Material 1

Supplementary Material 2

Supplementary Material 3

## Data Availability

No datasets were generated or analysed during the current study.

## Abbreviations

ATLS: Advanced Trauma Life Support
CRM: Crew Resource Management
ICC: Intraclass Correlation Coefficient
IRR: Inter-rater reliability
LEH: Local Emergency Hospitals
MTC: Major Trauma Centre
MMAT: Mixed Method Appraisal Tool
MHPTS: Mayo High Performance Teamwork Scale
NCEPOD: National Confidential Enquiry into Patient Outcome
NTS: Non-Technical Skills
NOTSS: Non-Technical Skills for Surgeons
PACT: Primary Assessment Completion Tool
PRISMA: Preferred Reporting Items for Systematic reviews and Meta-Analyses
TTLS-SHORT: Taxonomy of Trauma Leadership Skills–Shortened for Observation and Reflection in Training
TSAGT: Team Situation Awareness Global Assessment Technique
Team STEPPS: Team Strategies and Tools to Enhance Performance and Patient Safety
T- NOTECHs: Trauma NOn-TECHnical skills
TU: Trauma Units
VCR: Video Camera Recorders

## References

[1] Krug EG, Sharma GK, Lozano R. The global burden of injuries. Am J Public Health. 2000;90(4):523–6. 10.2105/AJPH.90.4.523. 10754963 PMC1446200

[2] Rossiter ND. Trauma—the forgotten pandemic? Int Orthop. 2022;46(1):3–11. 10.1007/s00264-021-05213-z. 34519840 PMC8438546

[3] Lendrum RA, Lockey DJ. Trauma system development. Anaesthesia. 2013;68(S1):30–9. 10.1111/anae.12049. 23210554

[4] Hakkenbrak NAG, Mikdad SY, Zuidema WP, Halm JA, Schoonmade LJ, Reijnders UJL, et al. Preventable death in trauma: a systematic review on definition and classification. Injury. 2021;52(10):2768–77. 10.1016/j.injury.2021.07.040. 34389167

[5] Craig HA, Lowe DJ, Khan A, Paton M, Gordon MW. Exploring the impact of traumatic injury on mortality: An analysis of the certified cause of death within one year of serious injury in the Scottish population. Injury. 2024;55(6):111470. 10.1016/j.injury.2024.111470. 38461710

[6] Choi J, Carlos G, Nassar AK, Knowlton LM, Spain DA. The impact of trauma systems on patient outcomes. Curr Probl Surg. 2021;58(1):100849. 10.1016/j.cpsurg.2020.100849. 33431134 PMC7286246

[7] Gabbe BJ, Simpson PM, Sutherland AM, Wolfe R, Fitzgerald MC, Judson R, et al. Improved functional outcomes for major trauma patients in a regionalized, inclusive trauma system. Ann Surg. 2012;255(6):1009–15. 10.1097/SLA.0b013e31824c4b91. 22584628

[8] Van Ditshuizen JC, Sewalt CA, Van Lieshout EM, Verhofstad MH, Den Hartog D. The association between level of trauma care and clinical outcome measures: a systematic review and meta-analysis. J Trauma Acute Care Surg. 2020;89(4):801–12. 10.1097/TA.0000000000002850. 33017136

[9] Flin R, Maran N. Basic concepts for crew resource management and non-technical skills. Best Pract Res Clin Anaesthesiol. 2015;29:27–39. 10.1016/j.bpa.2015.02.002. 25902464

[10] Vella MA, Zone A, Succar B, Cheng M, Maiga AW, Appelbaum RD, et al. Teamwork matters: The association between nontechnical skills and cardiac arrest in trauma patients presenting with hypotension. Surgery. 2024;175(6):1595–9. 10.1016/j.surg.2024.02.004. 38472080

[11] Cooper S, Wakelam A. Leadership of resuscitation teams: ‘Lighthouse Leadership’. Resuscitation. 1999;42(1):27–45. 10.1016/S0300-9572(99)00080-5. 10524729

[12] Xiao Y, Seagull FJ, Mackenzie CF, Klein K. Adaptive leadership in trauma resuscitation teams: a grounded theory approach to video analysis. Cogn Tech Work. 2004;6(3):158–64. 10.1007/s10111-004-0158-5.

[13] Yun S, Faraj S, Sims HP Jr. Contingent leadership and effectiveness of trauma resuscitation teams. J Appl Psychol. 2005;90(6):1288–99. 10.1037/0021-9010.90.6.1288. 16316282

[14] Ford K, Menchine M, Burner E, Arora S, Inaba K, Demetriades D, Yersin B. Leadership and teamwork in trauma and resuscitation. Western J Emerg Med. 2016;17(5):549–56. 10.5811/westjem.2016.7.29812. PMC5017838 27625718

[15] Härgestam M, Hultin M, Brulin C, Jacobsson M. Trauma team leaders’ non-verbal communication: video registration during trauma team training. Scand J Trauma Resusc Emerg Med. 2016;24:37. 10.1186/s13049-016-0230-7. 27015914 PMC4807541

[16] Abildgren L, Lebahn-Hadidi M, Mogensen CB, Toft P, Nielsen AB, Frandsen TF, et al. The effectiveness of improving healthcare teams’ human factor skills using simulation-based training: a systematic review. Adv Simul. 2022;7:12. 10.1186/s41077-022-00207-2. PMC9077986 35526061

[17] Burtscher MJ, Nussbeck FW, Sevdalis N, Gisin S, Manser T. Coordination and communication in healthcare action teams: The role of expertise. Swiss J Psychol. 2020;79(3–4):123–35. 10.1024/1421-0185/a000239.

[18] Vella MA, Dumas RP, Holena DN. Supporting the educational, research, and clinical care goals of the academic trauma center: Video review for trauma resuscitation. JAMA Surg. 2019;154(3):257–8. 10.1001/jamasurg.2018.5077. 30601875 PMC6753781

[19] Lowe DJ, Dewar A, Lloyd A, Edgar S, Clegg GR. Optimising clinical performance during resuscitation using video evaluation. Postgrad Med J. 2017;93(1102):449–53. 10.1136/postgradmedj-2016-134444. 27986970

[20] Page MJ, McKenzie JE, Bossuyt PM, Boutron I, Hoffmann TC, Mulrow CD, et al. The PRISMA 2020 statement: an updated guideline for reporting systematic reviews. BMJ. 2021;372:n71. 10.1136/bmj.n71. 33782057 PMC8005924

[21] Methley AM, Campbell S, Chew-Graham C, McNally R, Cheraghi-Sohi S. PICO, PICOS and SPIDER: a comparison study of specificity and sensitivity in three search tools for qualitative systematic reviews. BMC Health Serv Res. 2014;14:1–10. 10.1186/1472-6963-14-484. 25413154 PMC4310146

[22] Covidence systematic review software. Veritas Health Innovation, Melbourne, Australia. Available at www.covidence.org

[23] Hong QN, Fàbregues S, Bartlett G, Boardman F, Cargo M, Dagenais P, et al. Mixed methods Appraisal Tool (MMAT) Version 2018 for information professionals and researchers. Educ Inf. 2018;34(4):285–91. 10.3233/EFI-180218.

[24] Briggs A, Raja AS, Joyce MF, Yule SJ, Jiang W, Lipsitz SR, et al. The role of nontechnical skills in simulated trauma resuscitation. J Surg Educ. 2015;72(4):732–9. 10.1016/j.jsurg.2015.01.020. 25817012

[25] Crozier MS, Ting HY, Boone DC, O’Regan NB, Bandrauk N, Furey A, et al. Use of human patient simulation and validation of the Team Situation Awareness Global Assessment Technique (TSAGAT): a multidisciplinary team assessment tool in trauma education. J Surg Educ. 2015;72(1):156–63. 10.1016/j.jsurg.2014.06.012. 25441262

[26] Fernandez R, Rosenman ED, Olenick J, Misisco A, Brolliar SM, Chipman AK, et al. Simulation-based team leadership training improves team leadership during actual trauma resuscitations: a randomized controlled trial. Crit Care Med. 2020;48(1):73–82. 10.1097/CCM.0000000000004077. 31725441

[27] Hamilton NA, Kieninger AN, Woodhouse J, Freeman BD, Murray D, Klingensmith ME. Video review using a reliable evaluation metric improves team function in high-fidelity simulated trauma resuscitation. J Surg Educ. 2012;69(3):428–31. 10.1016/j.jsurg.2011.09.009. 22483149

[28] Leenstra NF, Jung OC, Cnossen F, Jaarsma ADC, Tulleken JE. Development and Evaluation of the Taxonomy of Trauma Leadership Skills–Shortened for Observation and Reflection in Training. Simul Healthc. 2021;16(1):37–45. 10.1097/SIH.0000000000000452. 32732816 PMC7850591

[29] Lorello GR, Hicks CM, Ahmed SA, Unger Z, Chandra D, Hayter MA. Mental practice: a simple tool to enhance team-based trauma resuscitation. CJEM. 2016;18(2):136–42. 10.1017/cem.2015.103. 25860822

[30] Ortiz FF, Yasmin M, Ramisha K, Rahul D, Blanda R, Marchand T, et al. Trauma Boot Camp: A Simulation-Based Pilot Study. Cureus. 2016;8(1):e456. 10.7759/cureus.456. 26929890 PMC4762770

[31] Rosser AA, Qadadha YM, Thompson RJ, Jung HS, Jung S. Measuring the impact of simulation debriefing on the practices of interprofessional trauma teams using natural language processing. Am J Surg. 2023;225(2):394–9. 10.1016/j.amjsurg.2022.10.021. 36207174

[32] Bhangu A, Notario L, Pinto RL, Pannell D, Thomas-Boaz W, Freedman C, et al. Closed loop communication in the trauma bay: identifying opportunities for team performance improvement through a video review analysis. CJEM. 2022;24(4):419–25. 10.1007/s43678-022-00305-6. 35412259 PMC9002216

[33] Dumas RP, Vella MA, Chreiman KC, Smith BP, Subramanian M, Maher Z, et al. Team assessment and decision making is associated with outcomes: a trauma video review analysis. J Surg Res. 2020;246:544–9. 10.1016/j.jss.2019.09.043. 31635832 PMC8406277

[34] Abd El-Shafy I, Delgado J, Akerman M, Bullaro F, Christopherson NA, Prince JM. Closed-loop communication improves task completion in paediatric trauma resuscitation. J Surg Educ. 2018;75(1):58–64. 10.1016/j.jsurg.2017.06.017. 28780315

[35] Hartwell V, Edmunds K, Elliott L, Williams B, Menk PT, Geis GL. Validity evidence for a team-leading assessment tool in paediatric emergency resuscitations using video review. AEM Educ Train. 2024;8(3):e10985. 10.1002/aet2.10985. 38693936 PMC11058601

[36] Henkel EB, Sampayo EM, Camp EA, Naik-Mathuria BJ, Chumpitazi CE, Wesson D, et al. Assessing leadership and clinical performance of pediatric emergency medicine providers during level 1 trauma resuscitations using video review. Pediatr Emerg Care. 2016. 10.1097/PEC.0000000000000966.

[37] Hoff WS, Reilly PM, Rotondo MF, DiGiacomo JC, Schwab CW. The importance of the command-physician in trauma resuscitation. J Trauma Acute Care Surg. 1997;43(5):772–7. 10.1097/00005373-199711000-00006. 9390488

[38] Hoyt DB, Shackford SR, Friland PH, Mackersie RC, Hansbrough JF, Wachtel TL, et al. Video recording trauma resuscitations: an effective teaching technique. J Trauma. 1988;28(4):435–40. 10.1097/00005373-198804000-00003. 3352005

[39] Lubbert PH, Kaasschieter EG, Hoorntje LE, Leenen LP. Video registration of trauma team performance in the emergency department: the results of a 2-year analysis in a level 1 trauma center. J Trauma. 2009;67(6):1412–20. 10.1097/TA.0b013e3181b83d16. 20009695

[40] Maiga AW, Vella MA, Appelbaum RD, Irlmeier R, Ye F, Holena DN, et al. Getting out of the bay faster: Assessing trauma team performance using trauma video review. J Trauma Acute Care Surg. 2023. 10.1097/TA.0000000000004037. 37880840 PMC12817889

[41] Rosenman ED, Bullard MJ, Jones KA, Welsh L, Brolliar SM, Levine BR, et al. Development and empirical testing of a novel team leadership assessment measure: a pilot study using simulated and live patient encounters. AEM Educ Train. 2019;3(2):163–71. 10.1002/aet2.10323. 31008428 PMC6457354

[42] Rosenman ED, Misisco A, Olenick J, Brolliar SM, Chipman AK, Vrablik MC, et al. Does team leader gender matter? A Bayesian reconciliation of leadership and patient care during trauma resuscitations. JACEP Open. 2021;2(1):e12348. 10.1002/emp2.12348. 33532754 PMC7823088

[43] Steinemann S, Berg B, Skinner A, DiTulio A, Anzelon K, Terada K, et al. In situ, multidisciplinary, simulation-based teamwork training improves early trauma care. J Surg Educ. 2011;68(6):472–7. 10.1016/j.jsurg.2011.05.009. 22000533

[44] Baker SP, O’Neill B, Haddon W Jr, Long WB. The injury severity score: a method for describing patients with multiple injuries and evaluating emergency care. J Trauma. 1974;14(3):187–96. 10.1097/00005373-197403000-00001. 4814394

[45] Bhangu A, Stevenson C, Szulewski A, MacDonald A, Nolan B. A scoping review of nontechnical skill assessment tools to evaluate trauma team performance. J Trauma Acute Care Surg. 2022;92(5):e81–91. 10.1097/TA.0000000000003504. 34908024

[46] Brogaard L, Hvidman L, Esberg G, Finer N, Hjorth-Hansen KR, Manser T, et al. Teamwork and adherence to guideline on newborn resuscitation—video review of neonatal interdisciplinary teams. Front Pediatr. 2022;10:828297. 10.3389/fped.2022.828297. 35265565 PMC8900704

[47] Mishra A, Catchpole K, McCulloch P, The Oxford NOTECHS. System: reliability and validity of a tool for measuring teamwork behaviour in the operating theatre. Qual Saf Health Care. 2009;18(2):104–8. 10.1136/qshc.2007.025379. 19342523

[48] Jacobsson M, Hargestam M, Hultin M, Brulin C. Flexible knowledge repertoires: communication by leaders in trauma teams. Scand J Trauma Resusc Emerg Med. 2012;20:44. 10.1186/1757-7241-20-44. 22747848 PMC3494569

[49] Likert R. The method of constructing an attitude scale. In: Seiler LH, editor. Scaling: A Sourcebook for Behavioral Scientists. Chicago: Aldine Publishing; 1970. pp. 233–42. (Book chapter).

