# Supplementary material for: Exploring tools used in the assessment of Trauma Team Leadership using video review: a systematic review
Source: Scand J Trauma Resusc Emerg Med. 2026 Jun 30;34:152. doi: 10.1186/s13049-026-01643-z (PMC13584448; doi:10.1186/s13049-026-01643-z)
Supplement: Supplementary file 1 [file 13049_2026_1643_MOESM1_ESM.docx]

|  | | **SCREENING QUESTIONS** | | **1. QUALITATIVE STUDIES** | | | | | **2. RANDOMIZED CONTROLLED TRIALS** | | | | | **3. NON-RANDOMIZED STUDIES** | | | | | **4. QUANTITATIVE DESCRIPTIVE STUDIES** | | | | | **5. MIXED METHODS STUDIES** | | | | | |
| --- | --- | --- | --- | --- | --- | --- | --- | --- | --- | --- | --- | --- | --- | --- | --- | --- | --- | --- | --- | --- | --- | --- | --- | --- | --- | --- | --- | --- | --- |
| **MMAT %age** | **First author. & Reference** | **S1. Are there clear research questions?** | **S2. Do the collected data allow to address the research questions?** | **1.1. Is the qualitative approach appropriate to answer the research question?** | **1.2. Are the qualitative data collection methods adequate to address the research question?** | **1.3. Are the findings adequately derived from the data?** | **1.4. Is the interpretation of results sufficiently substantiated by data?** | **1.5. Is there coherence between qualitative data sources, collection, analysis and interpretation?** | **2.1. Is randomization appropriately performed?** | **2.2. Are the groups comparable at baseline?** | **2.3. Are there complete outcome data?** | **2.4. Are outcome assessors blinded to the intervention provided?** | **2.5 Did the participants adhere to the assigned intervention?** | **3.1. Are the participants representative of the target population?** | **3.2. Are measurements appropriate regarding both the outcome and intervention (or exposure)?** | **3.3. Are there complete outcome data?** | **3.4. Are the confounders accounted for in the design and analysis?** | **3.5. During the study period, is the intervention administered (or exposure occurred) as intended?** | **4.1. Is the sampling strategy relevant to address the research question?** | **4.2. Is the sample representative of the target population?** | **4.3. Are the measurements appropriate?** | **4.4. Is the risk of nonresponse bias low?** | **4.5. Is the statistical analysis appropriate to answer the research question?** | **5.1. Is there an adequate rationale for using a mixed methods design to address the research question?** | **5.2. Are the different components of the study effectively integrated to answer the research question?** | **5.3. Are the outputs of the integration of qualitative and quantitative components adequately interpreted?** | **5.4. Are divergences and inconsistencies between quantitative and qualitative results adequately addressed?** | **5.5. Do the different components of the study adhere to the quality criteria of each tradition of the methods involved?** |  |
| 100% | **Bhangu (45)** |  |  |  |  |  |  |  |  |  |  |  |  |  |  |  |  |  |  |  |  |  |  |  |  |  |  |  |  |
| 80% | **Briggs (24)** |  |  |  |  |  |  |  |  |  |  |  |  |  |  |  |  |  |  |  |  |  |  |  |  |  |  |  |  |
| 80% | **Crozier. (25)** |  |  |  |  |  |  |  |  |  |  |  |  |  |  |  |  |  |  |  |  |  |  |  |  |  |  |  |  |
| 100% | **Dumas. (33)** |  |  |  |  |  |  |  |  |  |  |  |  |  |  |  |  |  |  |  |  |  |  |  |  |  |  |  |  |
| 100% | **El Shafy. (34)** |  |  |  |  |  |  |  |  |  |  |  |  |  |  |  |  |  |  |  |  |  |  |  |  |  |  |  |  |
| 100% | **Fernandez (26)** |  |  |  |  |  |  |  |  |  |  |  |  |  |  |  |  |  |  |  |  |  |  |  |  |  |  |  |  |
| 80% | **Hamilton. (27)** |  |  |  |  |  |  |  |  |  |  |  |  |  |  |  |  |  |  |  |  |  |  |  |  |  |  |  |  |
| 80% | **Hartwell. (35)** |  |  |  |  |  |  |  |  |  |  |  |  |  |  |  |  |  |  |  |  |  |  |  |  |  |  |  |  |
| 100% | **Henkel. (36)** |  |  |  |  |  |  |  |  |  |  |  |  |  |  |  |  |  |  |  |  |  |  |  |  |  |  |  |  |
| 100% | **Hoff. (37)** |  |  |  |  |  |  |  |  |  |  |  |  |  |  |  |  |  |  |  |  |  |  |  |  |  |  |  |  |
| 80% | **Hoyt. (38)** |  |  |  |  |  |  |  |  |  |  |  |  |  |  |  |  |  |  |  |  | Can't tell |  |  |  |  |  |  |  |
| 100% | **Leenstra. (28)** |  |  |  |  |  |  |  |  |  |  |  |  |  |  |  |  |  |  |  |  |  |  |  |  |  |  |  |  |
| 100% | **Lorello. (29)** |  |  |  |  |  |  |  |  |  |  |  |  |  |  |  |  |  |  |  |  |  |  |  |  |  |  |  |  |
| 80% | **Lubbert. (39)** |  |  |  |  |  |  |  |  |  |  |  |  |  |  |  |  |  |  |  |  |  |  |  |  |  |  |  |  |
| 100% | **Maiga. (40)** |  |  |  |  |  |  |  |  |  |  |  |  |  |  |  |  |  |  |  |  |  |  |  |  |  |  |  |  |
| 40% | **Ortiz Figue(30)** |  |  |  |  |  |  |  |  |  |  |  |  |  |  |  |  |  |  |  |  |  |  |  |  |  |  |  |  |
| 80% | **Rosenman(41)** |  |  |  |  |  |  |  |  |  |  |  |  |  |  |  |  |  |  |  |  |  |  |  |  |  |  |  |  |
| 80% | **Rosenman (42)** |  |  |  |  |  |  |  |  |  |  |  |  |  |  |  |  |  |  |  |  |  |  |  |  |  |  |  |  |
| 100% | **Rossier. (31)** |  |  |  |  |  |  |  |  |  |  |  |  |  |  |  |  |  |  |  |  |  |  |  |  |  |  |  |  |
| 100% | **Steinmann(43)** |  |  |  |  |  |  |  |  |  |  |  |  |  |  |  |  |  |  |  |  |  |  |  |  |  |  |  |  |
|  |  |  |  |  |  |  |  |  |  |  |  |  |  |  |  |  |  |  |  |  |  |  |  |  |  |  |  |  |  |
|  |  |  |  |  |  | **Yes** |  |  | **No** |  |  | **Can't tell** | |  |  |  |  |  |  |  |  |  |  |  |  |  |  |  |  |
