# Supplementary material for: Exploring tools used in the assessment of Trauma Team Leadership using video review: a systematic review
Source: Scand J Trauma Resusc Emerg Med. 2026 Jun 30;34:152. doi: 10.1186/s13049-026-01643-z (PMC13584448; doi:10.1186/s13049-026-01643-z)
Supplement: Supplementary file 2 [file 13049_2026_1643_MOESM2_ESM.docx]

**Appendix 1: Search Strategy**

Medline:

| **#** | **Query** | **Results from 18 Aug 2025** |
| --- | --- | --- |
| 1 | video*.mp. | 257,223 |
| 2 | leader*.mp. [mp=title, book title, abstract, original title, name of substance word, subject heading word, floating sub-heading word, keyword heading word, organism supplementary concept word, protocol supplementary concept word, rare disease supplementary concept word, unique identifier, synonyms, population supplementary concept word, anatomy supplementary concept word] | 141,501 |
| 3 | trauma*.mp. [mp=title, book title, abstract, original title, name of substance word, subject heading word, floating sub-heading word, keyword heading word, organism supplementary concept word, protocol supplementary concept word, rare disease supplementary concept word, unique identifier, synonyms, population supplementary concept word, anatomy supplementary concept word] | 563,540 |
| 4 | (review* or assessment* or evaluation* or judgment* or appraisal* or rating* or score* or tool* or taxonomy*).mp. [mp=title, book title, abstract, original title, name of substance word, subject heading word, floating sub-heading word, keyword heading word, organism supplementary concept word, protocol supplementary concept word, rare disease supplementary concept word, unique identifier, synonyms, population supplementary concept word, anatomy supplementary concept word] | 9,970,603 |
| 5 | 1 and 2 and 3 and 4 | 76 |
| 6 | limit 5 to English language | 75 |

Embase:

| **#** | **Query** | **Results from 18 Aug 2025** |
| --- | --- | --- |
| 1 | trauma*.mp. [mp=title, abstract, heading word, drug trade name, original title, device manufacturer, drug manufacturer, device trade name, keyword heading word, floating subheading word, candidate term word] | 780,176 |
| 2 | video*.mp. [mp=title, abstract, heading word, drug trade name, original title, device manufacturer, drug manufacturer, device trade name, keyword heading word, floating subheading word, candidate term word] | 333,522 |
| 3 | leader*.mp. [mp=title, abstract, heading word, drug trade name, original title, device manufacturer, drug manufacturer, device trade name, keyword heading word, floating subheading word, candidate term word] | 182,982 |
| 4 | (review* or assessment* or evaluation* or judgment* or appraisal* or rating* or score* or tool* or taxonomy*).mp. [mp=title, abstract, heading word, drug trade name, original title, device manufacturer, drug manufacturer, device trade name, keyword heading word, floating subheading word, candidate term word] | 14,027,264 |
| 5 | review*.mp. [mp=title, abstract, heading word, drug trade name, original title, device manufacturer, drug manufacturer, device trade name, keyword heading word, floating subheading word, candidate term word] | 6,119,084 |
| 6 | assessment*.mp. [mp=title, abstract, heading word, drug trade name, original title, device manufacturer, drug manufacturer, device trade name, keyword heading word, floating subheading word, candidate term word] | 4,477,271 |
| 7 | evaluation*.mp. [mp=title, abstract, heading word, drug trade name, original title, device manufacturer, drug manufacturer, device trade name, keyword heading word, floating subheading word, candidate term word] | 3,153,220 |
| 8 | 5 or 6 or 7 | (13,748,575) |
| 8 | 1 and 2 and 3 and 4 | 161 |
| 9 | limit 5 to English language | 160 |
