# Supplementary material for: Exploring tools used in the assessment of Trauma Team Leadership using video review: a systematic review
Source: Scand J Trauma Resusc Emerg Med. 2026 Jun 30;34:152. doi: 10.1186/s13049-026-01643-z (PMC13584448; doi:10.1186/s13049-026-01643-z)
Supplement: Supplementary file 3 [file 13049_2026_1643_MOESM3_ESM.docx]

**Appendix 2: Assessment Tools**

**T-NOTECHs**

**NOTSS**

**Mayo**

**Team STEPPS**

**Leadership Behaviour DQ Adapted**

**Novel team leadership assessment tool**

**EM Resuscitation Team Leadership Measure**
